# The activation of the metabolic oxaloacetate-pyruvate axis restores influenza A virus replication during impaired glycolysis

**DOI:** 10.1186/s12985-026-03201-6

**Published:** 2026-05-24

**Authors:** Chiara Robin Bojarzyn, Nadine Bieß, Matthias Behrens, Hans-Ulrich Humpf, Stephan Ludwig, Eike R. Hrincius

**Affiliations:** 1 Institute of Virology Muenster (IVM), University of Muenster, Von-Esmarch-Straße 56, 48149 Muenster, Germany; 2 Institute of Food Chemistry, University of Muenster, Corrensstraße 45, 48149 Muenster, Germany

## Abstract

Viruses strongly depend on the host cell for efficient replication and influenza A virus (IAV) amongst others also lead to remarkable changes of the host cell metabolism. The restriction of virus replication through suppression of glucose metabolism has already been described. In addition to glycolysis and glutaminolysis, viral replication also relies on the tricarboxylic acid (TCA) cycle. So far, the metabolic key intermediate of the TCA cycle, oxaloacetate (OAA), is described to enhance glycolysis and respiration flux rates under metabolic stress conditions. However, the mode of action of the metabolic fuel intermediate OAA in direct relation to influenza viral growth under strong glycolysis inhibition remains unclear. As the TCA cycle acts as a central metabolic hub linking all major metabolic pathways, the effects of OAA under glycolysis inhibition were examined in greater detail in this study. We aimed to get a better understanding of metabolic host-virus interactions and to analyze the effects of metabolic fueling intermediates on IAV replication under glycolysis inhibition. We inhibited glycolysis and supplemented IAV infected cells with the metabolic fueling intermediate OAA. Inhibition of glycolysis led to a statistically significant reduction of viral titers while OAA addition reversed the antiviral effects such as reduced viral protein accumulation, viral titers, and vRNA expression. In line with previous studies, we showed that mannose, which is closely connected to glycolysis, circumvents the virus restricting effects of glycolysis inhibition. Moreover, we demonstrated that supplementation of mannose or OAA led to a roughly comparable replication recovery under strong inhibition of glycolysis. Furthermore, mass spectrometry-based metabolomics data revealed a strong accumulation of pyruvate in OAA supplemented samples. Finally, comparing OAA and pyruvate rescuing capacities of IAV growth under glycolysis inhibition tended to show similar activities for both metabolites arguing that OAA mediated IAV rescue is achieved through its conversion to pyruvate. Summarizing, our data indicate that the TCA cycle intermediate OAA has virus supporting effects as it reversed the antiviral effects of glycolysis inhibition.

**Supplementary Information:**

The online version contains supplementary material available at 10.1186/s12985-026-03201-6.

## Background

Influenza viruses (IVs) continue to pose a major threat to human health and represent a challenge to the global healthcare system. Influenza A virus (IAV) causes acute respiratory infections in humans across all age groups and can lead to fatal outcomes in severe cases [1, 2]. Moreover, it is known for its annually recurring epidemics, and its potential to cause pandemics, which have been characterized by global spread with high casualty numbers [3, 4]. Currently, vaccination is the most common way of preventing an IAV infection. However, the vaccination must be repeated annually due to the constant antigenic drift of the virus [5]. Direct acting antiviral drugs, such as Tamiflu or Baloxavir are available, however they are only efficient if applied very early after infection and are prone to the emergence of resistance [6]. Therefore, novel antiviral therapeutic strategies are required, and one promising approach is host-directed antiviral therapies that for example interfere with the metabolism of the host cell. To identify new promising targets for future host-directed antiviral therapies, we must first gain fundamental knowledge about the metabolic changes induced by IAV and the molecular needs of IAV regarding cellular metabolites. This is why the aim of this study was to examine the impact of metabolic treatments on the IAV life cycle and thus to develop a more fundamental understanding of metabolic intervention potentials.

As viruses are obligate intracellular parasites, they strongly depend on host cell metabolism to replicate successfully. We and others have demonstrated that IAV strongly affects certain metabolic pathways and cellular functions [7, 8, 9, 10], but comprehensive knowledge of how cellular metabolism impacts the IAV life cycle remains limited. In general, cellular metabolism is a highly regulated and complex network of biochemical reactions that transform metabolic products, to ensure the survival of living organisms [11]. Although some potential metabolic targets are already known [8, 12], a better understanding of the nature of metabolic changes caused by IAV infections is of great importance. A common consequence of a viral infection is an elevated glucose metabolism [7, 8, 9]. Most virus-infected cells generate energy through aerobic glycolysis, an effect that is also described for most cancer cells, the so-called Warburg effect [13, 14]. During IAV infections, the virus has been shown to enhance glucose uptake and elevate extracellular lactate levels in host cells [9]. Accordingly, IAV is reported to increase glycolysis, which already has been shown in various studies [10, 15]. Inhibition of glycolysis using 2-deoxy-D-glucose (2-DG), led to an impairment of IAV reproduction [10, 15, 16]. When 2-DG was applied, IAV viral titers and the accumulation of genomic vRNA were statistically significantly reduced in a dose-dependent manner while the phase of viral mRNA synthesis was prolonged [10]. These data suggest that reduced viral replication upon glycolytic interference occurs mainly due to an impairment of the dynamic regulation of the viral polymerase [10]. Furthermore, it has been demonstrated that IAV replication is also altering other metabolic pathways, including pentose phosphate pathway (PPP), fatty acid synthesis (FAS), tricarboxylic acid cycle (TCA), glutaminolysis or oxidative phosphorylation (OXPHOS) [8, 9, 17, 18]. In turn, inhibition of all these different metabolic pathways led to statistically significantly reduced viral titers [19]. Surprisingly, the inhibition of the fatty acid synthesis (FAS), glutaminolysis and oxidative phosphorylation (OXPHOS) resulted in reduced vRNA levels. Moreover, the inhibition of these pathways caused an unbalance of the cellular glycolysis and respiration network impairing the dynamic regulation of the viral polymerase [19]. Since glycolysis is important for successful IAV replication, the impact of the subsequent metabolic intermediates of glycolysis were of great importance in this research. Studies have shown that glycolysis can be fueled not only by glucose but also by various other metabolites, entering the pathway through an interconnected metabolic network. For instance, glycolysis is closely connected to the mannose metabolism and the glycolytic intermediate fructose-6-phosphate (F-6-P) and mannose-6-phosphate (M-6-P) can be converted to each other by mannose-6-phosphate isomerase (MPI). Consistent with this direct connection, mannose was confirmed to circumvent the IAV inhibitory effect of 2-DG by refueling glycolysis [10]. A better understanding of metabolic changes induced by IAV infections is of considerable relevance, which is why this work includes the investigation of metabolic fueling intermediates in the context of IAV infection. For viral replication, TCA cycle metabolites are crucial for energy production, pro-inflammatory and anti-inflammatory homeostasis [20, 21]^,^. The TCA cycle proceeds following glycolysis under aerobic conditions. TCA cycle intermediates fulfill diverse cellular functions, including regulation of cellular immunity, fatty acid synthesis or insulin secretion [22].

In this study, we targeted glycolysis and supplemented with a TCA cycle intermediate, under the assumption that restoring downstream TCA cycle activity would compensate for glycolytic inhibition and re-boost viral growth. We intended to analyze the metabolic fueling intermediate OAA, a key metabolite of the TCA cycle, which takes place within the mitochondria. In brief, pyruvate, the end-product of glycolysis, is imported into the mitochondria via mitochondrial pyruvate carrier (MPC) to be oxidized into acetyl-coenzyme-A (acetyl-CoA) [23]. The TCA cycle begins with the condensation of OAA combined with acetyl-CoA to form citrate, a reaction which is catalyzed by citrate synthase. Moreover, the generated energy from the TCA cycle reactions is stored in FADH_2_ and NADH, which undergo oxidation by the electron transport chain, for the synthesis of ATP. OAA is involved in different metabolic pathways to maintain the bioenergetic flow. It is known to be associated with amino acid synthesis, fatty acid synthesis, and gluconeogenesis. In addition, OAA supplementation to cells can support or enhance glycolysis and respiration flux rates under metabolic stress conditions [24, 25]. This observation is of particular interest to this work, as the effects of OAA on IAV infection in direct relation to glycolysis inhibition have not yet been investigated. Although several studies have examined that the host metabolism of infected IAV cells is modified, the exact underlying mechanism of how the viral polymerase activity is influenced by the host metabolism is largely unknown.

## Materials and methods

### Cell lines and viruses

Human adenocarcinomic alveolar basal epithelial cells (A549, American type culture collection (ATCC®), CCL-185™) were cultured in Dulbecco’s modified Eagle’s medium high glucose (DMEM, Sigma-Aldrich, D5796) supplemented with 10% fetal bovine serum (FBS). Madin-Darby canine kidney (MDCK) II cells (Institute of Virology, University of Muenster, Germany) were maintained in minimum essential medium (MEM, Sigma-Aldrich, M4655), supplemented with 10% FBS. The different cell lines were incubated under conditions of 37 °C and 5% CO_2_. Recombinant mouse-adapted A/Seal/Massachusetts/1/80 H7N7 (SC35M) was propagated in MDCK II cells.

### Infection and inhibitor treatment

For viral infection, cells were phosphate-buffered saline (PBS) washed and afterwards incubated with virus at the desired MOI in infection PBS supplemented with 0.2% bovine serum albumin (BSA), 1 mM MgCl_2_, 0.9 mM CaCl_2_, 100 U/mL penicillin and 0.1 mg/mL streptomycin at 37 °C and 5% CO_2_. After 30 min, cells were washed with PBS and incubated with the indicated concentrations of inhibitor and or supplement in infection DMEM media, which was supplemented with 0.2% bovine serum albumin (BSA), 100 U/mL penicillin and 0.1 mg/mL streptomycin, 25 mM D-glucose, and 2 mM L-glutamine. The virus supernatants or cell lysates were frozen at − 80 °C until experimental analysis. 2-DG (Sigma Aldrich, D8375), mannose (Sigma Aldrich, M6020), OAA (Sigma Aldrich, O4126), sodium pyruvate (Merck, P5280) were dissolved in water to 1 M stock solutions.

### Focus-forming assay

After infection, cell supernatant was collected and frozen at − 80 °C. MDCK II cells were infected with ten-fold serial dilutions of respective supernatant for 30 min. These dilutions were prepared in infection PBS. Then, avicel-overlay (containing one volume of 2.5% avicel solution and one volume of 2x MEM (containing 0.21% BSA, 0.21% NaHCO_3_, 1 mM MgCl_2_, 0.01% DEAE-dextran, 0.9 mM CaCl_2_, 100 U/ml penicillin, 0.1 mg/ml streptomycin) and 2 mM L-glutamine) was added to the virus inoculum and incubated for 24 hs at 37 °C and 5% CO_2_. After 24 hs of incubation time, the Avicel containing overlay was washed off with PBS. Afterwards, cells were covered with ice-cold methanol (100%) and incubated for 10 min at 4 °C for fixation. The cells were washed three times with PBS and mouse anti influenza NP hybridoma supernatant (ATCC HB-65, clone H16-L10-4R5) was applied for 1 h at room temperature. After washing the cells three times with PBS containing 0.05% Tween-20, the fluorophore-labelled secondary antibody IRDye^®^ 680RD Donkey anti-Mouse (LI-COR, 92668072) was added for 1 h at room temperature. The virus infected foci were counted using the Celigo Image Cytometer (Nexcelom/Perkin Elmer Inc., Waltham, MA, USA). Virus titers were calculated as foci-forming units per milliliter (FFU/ml). Representative pictures obtained during the assays and used for calculations are shown in Fig. S1.

### Reverse transcription and quantitative real-time PCR

For quantification of viral mRNA and vRNA expression levels, total RNA was isolated using the RNeasy® Plus Mini Kit (Qiagen). This was done according to the manufacturer’s protocol. Followed by reverse transcription using the RevertAid™ H Minus Reverse Transcriptase (Thermo Fisher Scientific) with oligo(dT) primers (Eurofins Genomics) to synthesize cDNA from mRNA or a fluA uni12 forward primer (5’-AGCAAAAGCAGG-3’) (Eurofins Genomics) to transcribe vRNA according to the manufacturer’s instructions. The cDNA was used for real-time qPCR performed with a LightCycler 480 II (Roche) and Brilliant III SYBRⓇ Green (Agilent) according to the manufacturer’s manual. The following primers were used during qPCR: influenza matrix protein M1 forward (5’-AGA TGA GTC TTC TAA CCG AGG TCG-3’) and reverse (5’-TGC AAA AAC ATC TTC AAG TCT CTG-3’), human Mx1 forward (5’-GTT TCC GAA GTG GAC ATC GCA-3’) and reverse (5’-GAA GGG CAA CTC CTG ACA GT-3’), DDX58 forward (5’-CCT ACC TAC ATC CTG AGC TAC AT-3’) and reverse (5’-TCT AGG GCA TCC AAA AAG CCA-3’), and human glyceraldehyde 3-phosphate dehydrogenase (GAPDH) forward (5’-GCA AAT TCC ATG GCA CCG T-3’) and reverse (5’-GCC CCA CTT GAT TTT GGA GG-3’) (Eurofins Genomics). GAPDH, functioned as a housekeeping gene and was used for the normalization. The relative n-fold was obtained based on the 2-ΔΔCT method [26].

### Strand specific quantitative real-time RT-PCR

After infection, total RNA was purified as previously described in 2.4. The total RNA was reverse transcribed using Maxima Reverse Transcriptase (Thermo Fisher Scientific) according to the manufacturer’s protocol. For detection of viral mRNA and vRNA, specific mRNA or vRNA tag primers (Eurofins Genomics) were used as reported [27]. The PB2 primer sequences used for qPCR were identical to those described by Kleinehr et al. [10].

### Western blot

After 8 h of infection, cells were lysed with pre-cooled radioimmunoprecipitation assay (RIPA) buffer (25 mM Tris-HCl pH 8, 137 mM NaCl, 10% glycerol, 0.1% SDS, 0.5% NaDOC, 1% NP-40, 2 mM EDTA pH 8, 200 µM Pefabloc®, 5 µg/mL aprotinin, 5 µg/mL leupeptin, 1 mM sodium orthovanadate and 5 mM benzamidine). Unified samples were centrifuged for 10 min to remove cell debris and relative protein concentration of samples was determined via Bradford assay. Samples were adjusted to the same protein concentration, mixed with the appropriate amount of Laemmli sample buffer (10% SDS, 50% glycerol, 25% 2-mercaptoethanol, 0.01% bromophenol blue, 312 mM of 1 M Tris (pH 6.8)) and separated by SDS-PAGE. For western blot analysis, the proteins were transferred to a nitrocellulose membrane. Subsequently, proteins were detected using the following primary antibodies: vinculin (mouse, monoclonal, Merck, MAB3574), M1 (mouse, monoclonal, Biorad, MCA401), NP (rabbit, polyclonal, GeneTex, GTX125989), NS1 (rabbit, polyclonal, GeneTex, GTX125990) and PA (rabbit, polyclonal, GeneTex, GTX125932). Vinculin served as the loading control. The following IRDye secondary antibodies (Li-COR)) labelled with near-infrared (NIR) fluorescent dyes were used for direct, nonenzymatic detection of primary antibodies: IRDye^®^ 680RD Donkey anti-Mouse (LI-COR, 92668072), IRDye^®^ 680RD Donkey anti-Rabbit (LI-COR, 92668073), IRDye^®^ 800CW Donkey anti-Mouse (LI-COR, 92632212) and IRDye^®^ 800CW Donkey anti-Rabbit (LI-COR, 92632213). For protein band detection, images were visualized with an ODYSSEYⓇ F_C_ Imaging System (LI-COR). Relevant bands were cropped and grouped together. Band intensity was quantified using Image Studio lite quantification software (LI-COR). Original blots are shown in Fig. S2.

### Metabolic profiling by HILIC-MS/MS

Metabolic profiling analysis was done as described before [10]. In brief, A549 cells were infected with SC35M at an MOI of 5 or mock infected in infection PBS supplemented with 0.2% bovine serum albumin (BSA), 1 mM MgCl_2_, 0.9 mM CaCl_2_, 100 U/mL penicillin and 0.1 mg/mL streptomycin at 37 °C and 5% CO_2_ for 30 min. Then, cells were incubated with the indicated concentrations of inhibitor and or supplement in infection DMEM media for a total of 8 h. Afterwards, cells were washed twice with PBS and pre-cooled acetonitrile (ACN)/water (4 + 1, v/v) including D-phenylglycine (50 µM) as internal standard for metabolic quenching. Cells were detached with a sterile cell scraper, dishes were washed with additional ACN/water (4 + 1, v/v) and pooled with the respective cell samples. Samples were stored at -80 °C until chromatographic and mass spectrometric analysis [10, 28].

### Real-time monitoring of cellular metabolism via seahorse XF HS mini analyzer

For Seahorse Analyzer experiments, A549 cells were seeded on a Seahorse XFp cell culture miniplate (Agilent) and incubated for 24 h at 37 °C and 5% CO_2_. To prepare for analysis, the Seahorse XFp extracellular flux cartridge (Agilent) was incubated in XF calibrant solution (Agilent) for 24 h at 37 °C in a non-CO_2_ incubator. After 24 h, cells were rinsed with PBS and then incubated using Seahorse XF DMEM medium supplemented with 2 mM L-glutamine and 25 mM D-glucose. After measuring basal ECAR, OCR, and GlycoPER, inhibitors or solutions were injected and the parameters were continuously monitored for 3.5 h using the Seahorse XF HS Mini Analyzer (Agilent).

### Statistical analysis

Statistical analyses were performed via GraphPad Prism 9 (Version 9.5.1, GraphPad Software, LLC).

## Results

### Oxaloacetate treatment led to a rescue of the inhibitory effect of 2-DG on viral growth

**Fig. 1:**
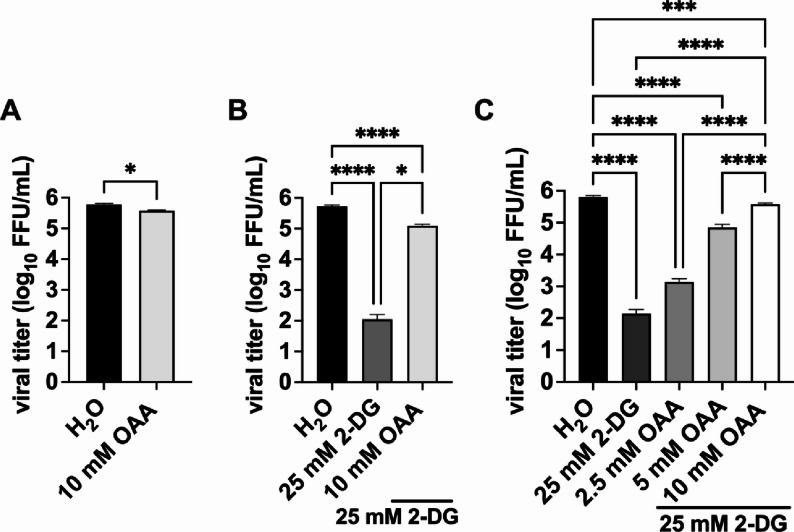
Oxaloacetate supplementation dose-dependently rescued viral titers under 2-DG-mediated glycolysis inhibition. A549 cells were infected with SC35M at an MOI of 0.001 for 30 min. Subsequently, the infected cells were treated either with 10 mM oxaloacetate (OAA) **(A)**, or with 25 mM 2-DG and the indicated concentration of OAA **(B**,** C)**, or the solvent control water in DMEM infection media for a total of 24 h. After 24 h, viral titers (FFU/mL) were determined via FFA. Depicted are the means ± SD of three independent experiments with biological triplicates per condition and experiment. Statistical significances were determined via unpaired Welch’s t test **(A)**, or via ordinary ANOVA with Tukey’s correction **(B**,** C)** comparing all samples with each other. *P*-values are indicated as follows: < 0.05 = *, < 0.01 = **, < 0.001 = ***, < 0.0001 = ****

Since viruses are highly dependent on the host cell for efficient replication, we wanted to gain a better understanding of metabolic interactions between the virus and its host and examined the effects of metabolic fueling intermediates on IAV replication under glycolysis inhibition. Our first aim was to test whether the treatment of cells with the TCA cycle intermediate oxaloacetate (OAA) alone affects IAV propagation. Hence, we used focus forming assays (FFA) to examine the number of newly produced infectious IAV particles of A549 cells 24 h post infection (hpi) with the mouse-adapted recombinant IAV strain A/Seal/Massachusetts/1/80 H7N7 (SC35M) at a multiplicity of infection (MOI) of 0.001. First, we observed that the supplementation of OAA into tissue culture media after the initial infection had only a marginal effect on viral titers (Fig. 1A). Furthermore, we showed that the number of newly produced infectious IAV particles decreased statistically significantly when 25 mM 2-DG was applied (Fig. 1B).

Next, we aimed to characterize the effects of OAA during glycolysis inhibition on virus growth. Interestingly, we almost completely rescued viral titers with OAA supplementation under 2-DG treatment (Fig. 1B) showing that the 2-DG-mediated decrease in virus growth was almost completely reversed by OAA addition. To verify this effect in more detail, we compared different OAA concentrations under glycolysis inhibition regarding their rescuing capacities of IAV growth. As before, the addition of the glycolysis inhibitor 2-DG led to a statistically significant decrease of viral titers (Fig. 1C) and the number of newly produced infectious IAV particles increased statistically significantly in a dose-dependent manner when OAA was applied. Summing up, these data demonstrate that OAA almost completely reversed the inhibitory effect of 2-DG on viral growth.

### Oxaloacetate and mannose were equally potent to restore IAV replication under strong glycolysis inhibition

**Fig. 2:**
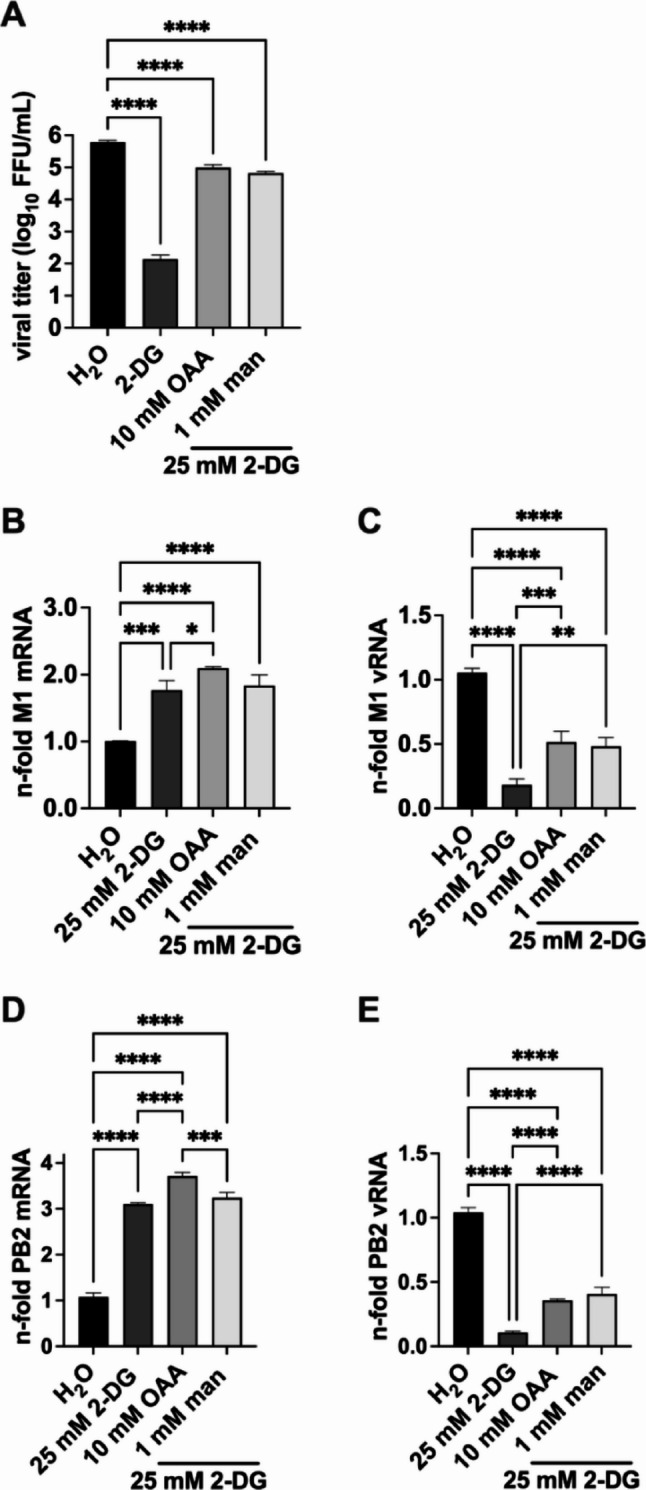
Oxaloacetate and mannose rescued viral growth under strong glycolysis inhibition. 24 h after seeding, A549 cells were infected with SC35M at an MOI of 0.001 **(A)** or 5 **(B-E)** for 30 min. Then, the infected cells were treated either with 25 mM 2-DG, or 2-DG combined with 10 mM oxaloacetate (OAA), or 1 mM mannose (man), or the solvent control water in DMEM infection media. For focus forming assay, viral titers (FFU/mL) were determined after a total infection of 24 h **(A)**. For real-time qPCR, cells were lysed after 8 h of infection **(B-E)** followed by RNA isolation. Then, cDNA was synthesized using either oligo(dT) primers to transcribe mRNA **(B)**, fluA uni12 primers to transcribe vRNA **(C)**, or specific primers to transcribe mRNA and vRNA of the SC35M gene segment 1 (PB2) [27] **(D**,** E)**. Realtime qPCR was performed with two technical replicates per sample and values of the treated samples were normalized to the water control. In case of mRNA detection, all results were additionally normalized to a GAPDH control. Depicted are the means ± SD of three independent experiments with biological triplicates per condition and experiment. Statistical significances were determined via ordinary ANOVA with Tukey’s correction **(A-E)** where all samples were compared to each other. P-values are indicated as follows: < 0.05 = *, < 0.01 = **, < 0.001 = ***, < 0.0001 = ****

To understand the OAA-mediated rescue of IAV replication during glycolysis inhibition in more detail, we investigated its effect on viral gene transcription and genome replication. In addition, we compared the rescuing capabilities of OAA with mannose, as the mannose metabolism is described to be directly linked to glycolysis by the enzyme mannose-6-phosphate isomerase (MPI) [10, 29] and it was already shown that mannose circumvents the virus-restricting impact of 2-DG by refueling glycolysis [10]. By performing focus forming assays we detected an increased number of newly produced infectious IAV particles with the supplementation of OAA during 2-DG treatment (Fig. 2A). These results were in accordance with the previous data (Fig. 1B, C). Furthermore, we showed that the addition of mannose restored the antiviral effect of 2-DG on viral titers as well and interestingly, the OAA- and the mannose-mediated rescue were equally potent.

**Fig. 3:**
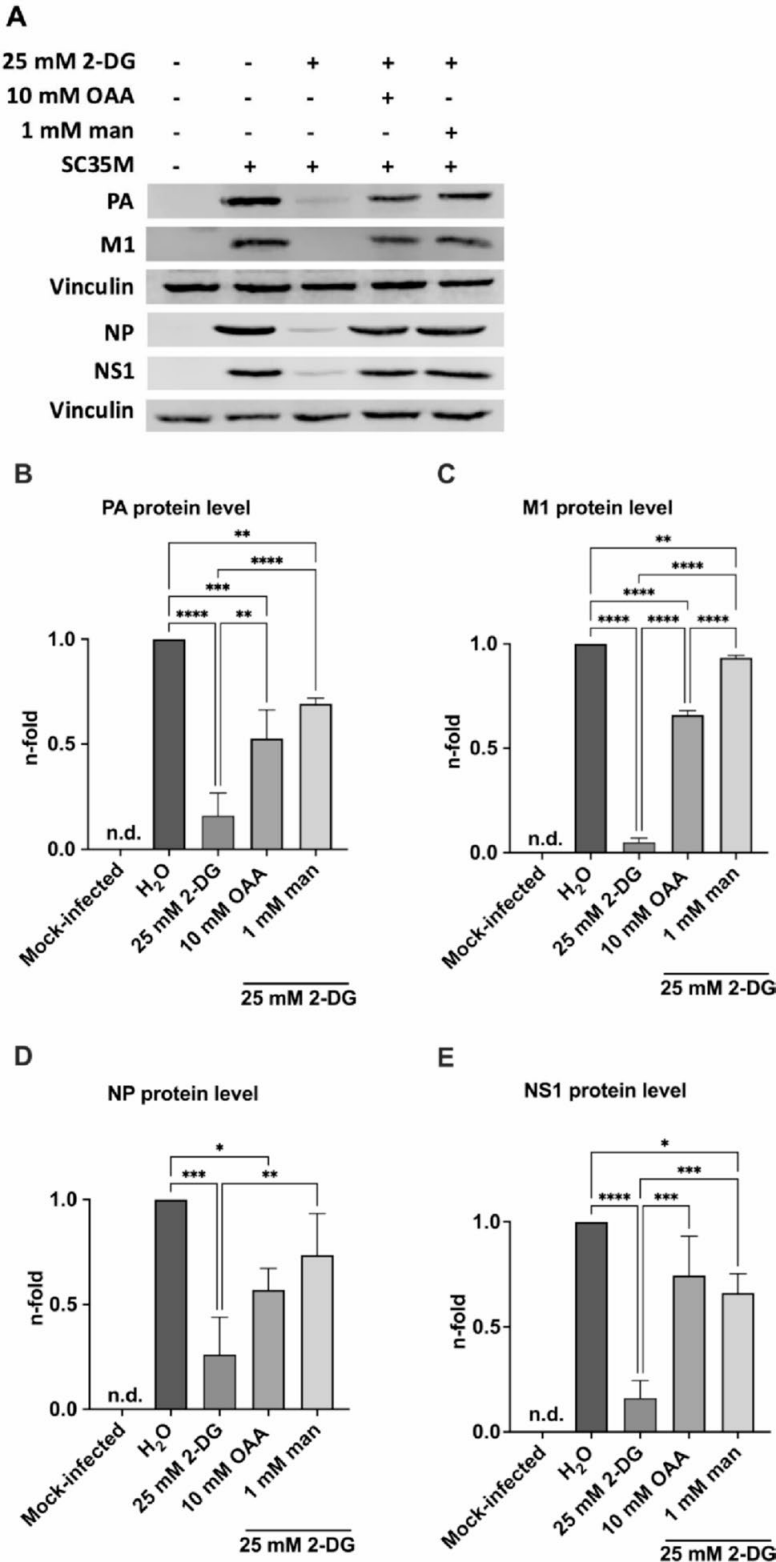
Oxaloacetate and mannose increased viral protein accumulation under glycolysis inhibition within a single viral replication cycle. A549 cells were mock-infected or infected with SC35M at an MOI of 5 for 30 min. Afterwards, the infected cells were treated either with 25 mM 2-DG, or 2-DG combined with 10 mM oxaloacetate (OAA), or 1 mM mannose (man), or the solvent control water in DMEM infection media for a total of 8 h **(A-E)**. Protein lysates were harvested, equal amounts of protein were separated via SDS-PAGE and subjected to Western Blot analysis. Visualization was done using primary antibodies against PA (rabbit), M1 (mouse), NP (rabbit), NS1 (rabbit), and vinculin (mouse) and fluorescence-labelled secondary antibodies. Western Blot images were cropped and representative blots out of three independent experiments are shown. Protein signals that were not detectable are indicated as n.d. Densitometric quantifications of the viral protein bands were normalized to the loading control vinculin and the infected but untreated sample **(B-E)** to quantify protein accumulation. The means ± SD of three independent experiments with biological triplicates per condition and experiment are shown. Statistical significances were determined via ordinary ANOVA with Tukey’s correction **(B-E)** where all samples were compared to each other. P-values are indicated as follows: < 0.05 = *, < 0.01 = **, < 0.001 = ***, < 0.0001 = ****

In addition, we also tested the effects of OAA treatment on the accumulation of viral mRNA and vRNA through real-time qPCR. As a readout of vRNA detection, we used M1 values representative of vRNA segment 7, as well as PB2 values representative of vRNA segment 1. While the treatment of 2-DG led to an increase of the viral mRNA levels, vRNA levels were decreased after a single replication cycle of 8 h (Fig. 2B-E). These findings were consistent with our previously published data showing that glycolysis inhibition resulted in elevated viral mRNA levels but decreases the amount of vRNA within a single viral life cycle [10]. Furthermore, we observed slightly increased viral mRNA levels after the treatment with OAA under strong glycolysis inhibition. More important, upon treatment with OAA combined with 2-DG, there were increased vRNA levels compared to the only 2-DG treatment within one replication cycle. The results for vRNA levels were very similar for segment 1 and segment 7. Moreover, these effects were equally effective to the mannose mediated rescue (Fig. 2B-E). To sum up, vRNA levels were clearly reduced by 2-DG and partly rescued by OAA and mannose, whereas viral mRNA levels were generally elevated. Considering the statistically significant rescue of IAV replication by OAA during strong glycolysis inhibition, we now aimed to explore possible effects on the production of IAV viral proteins after a single replication cycle of 8 h as well. Therefore, we analyzed potential changes in the accumulation of the IAV proteins PA, M1, NP, and NS1 after 8 hpi (Fig. 3).

In line with the increased viral titers and vRNA levels, the accumulation of viral proteins was statistically significantly increased in the presence of OAA under strong glycolysis inhibition (Fig. 3). Within a single replication cycle, we detected equally potent strength of the OAA- and mannose-mediated rescue of 2-DG treatment mediated diminishing of protein band intensities. Taken together, the results confirmed the dependency of IAV on metabolic fuel intermediates of the host cell, as we proved that OAA reversed the inhibitory effect of strong glycolysis inhibition on viral growth, viral vRNA and viral protein accumulation.

### Oxaloacetate supplementation resulted in a marked accumulation of pyruvate

**Fig. 4:**
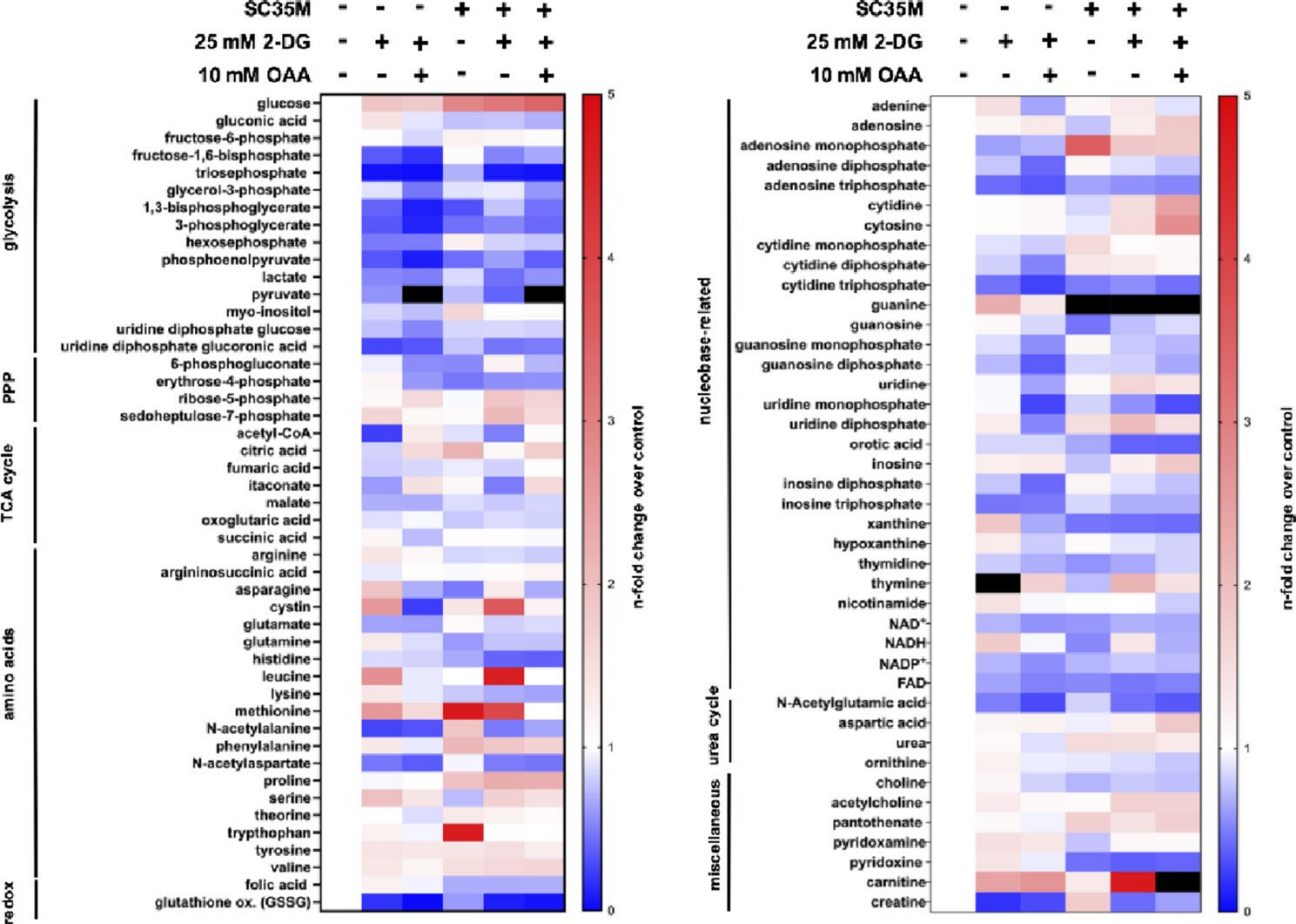
Mass spectrometry-based metabolomics revealed elevated pyruvate levels in oxaloacetate supplemented samples. A549 cells were mock-infected or infected with SC35M at an MOI of 5. Afterwards, cells were treated with 25 mM 2-DG alone, in combination with 10 mM oxaloacetate (OAA), or the solvent control water in DMEM infection media for a total of 8 h. Then, the metabolic activity of the samples was quenched and metabolites were quantified relative to the control via HILIC-MS/MS. All data were normalized to the uninfected and untreated control. Darker red shades represent higher levels, while darker blue shades indicate a lower n-fold change of the different metabolites compared to control. Black shades indicate a metabolite increase greater than fivefold relative to the control. The means of three independent experiments with biological triplicates per condition and experiment are shown. The heatmap should be interpreted independently of statistical significance. The n-folds and p-values are presented in Fig. S3

To gain a broader understanding of metabolic alterations induced by IAV and treatment with OAA under glycolysis inhibition we analyzed the metabolic profile via hydrophilic interaction liquid chromatography (HILIC) coupled to tandem mass spectrometry (MS/MS) (Fig. 4). For this experimental setup, A549 cells were infected with SC35M at an MOI of 5 for 8 h with the addition of 2-DG, OAA, or its combination.

This mass spectrometry-based analysis allows quantification of a broad spectrum of metabolites, including amino acids, intermediates of urea cycle, TCA cycle, glycolysis, and the PPP. Among amino acids, such as arginine, cystine, and serine, we observed no clear trend of changes when comparing the different treatments or between infected and uninfected cells. In the following, we focused on those metabolites that play a role in cell metabolism, as this work provides fundamental knowledge about the interaction between IAV and host cell metabolism. As seen before [7, 8, 9, 10], glucose levels were statistically significantly increased in infected cells compared to uninfected cells (Fig. 4) (Fig S3). The highest glucose levels were measured in the infected samples treated with 2-DG and OAA. When 2-DG was applied, most of the glycolytic intermediates were decreased independent of infection. We observed no marked differences for the measured glycolysis intermediates between the 2-DG treatment alone and the combined treatment with OAA and 2-DG, with the exception of pyruvate. Surprisingly, the addition of 10 mM OAA to 2-DG treated cells led to a massive statistically significant increase of the pyruvate levels independent of viral infections (Fig S3). A comparison of TCA cycle intermediates from uninfected and infected cells showed that most of the metabolites were slightly decreased or unchanged in infected cells, with the exception of citric acid, which was statistically significantly increased. The majority of the TCA cycle intermediates, especially acetyl-CoA, were decreased when 2-DG was applied. Although it was not statistically significant, it was interestingly, that the addition of OAA led to an increase of certain TCA cycle intermediate levels, for example acetyl-CoA, compared to 2-DG treatment alone.

All in all, the mass spectrometry-based metabolomics data revealed changes in the metabolic profile due to the treatments with a strong accumulation of pyruvate in OAA supplemented samples.

### Pyruvate and OAA comparably rescued IAV replication under strong glycolysis inhibition

**Fig. 5:**
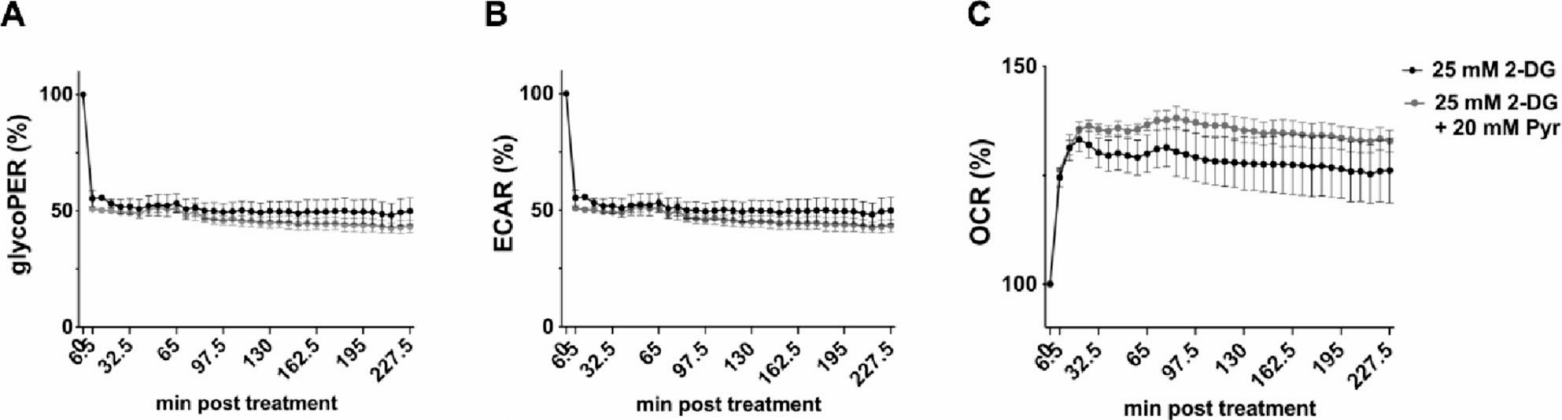
Pyruvate increased oxygen consumption rate under glycolysis inhibition. 24 hs after seeding A549 cells, cells were incubated in XF DMEM medium supplemented with 25 mM glucose and 2 mM L-glutamine for 1 h at 37 °C in a non-CO_2_ incubator. During the measurement the cells were treated either with 25 mM 2-DG, or the indicated concentrations of pyruvate in combination with 25 mM 2-DG. The glycolytic proton efflux rate (glycoPER) (**A**), the extracellular consumption rate (ECAR) (**B**), and the oxygen consumption rate (OCR) (**C**) were measured in real time with a Seahorse Analyzer. Depicted are the means ± SD of four independent experiments with biological triplicates per condition and experiment. Statistical significances were determined via ordinary two-way ANOVA with Sidak’s correction, comparing all the pyruvate treated samples of a time point to its respective control. P-values are indicated as follows: < 0.05 = *, < 0.01 = **, < 0.001 = ***, < 0.0001 = ****

To further investigate the effect of massive pyruvate accumulation in OAA treated samples, we analyzed the effect of elevated pyruvate levels during OAA supplementation in more detail. Consistent with the markedly increased pyruvate levels observed in the OAA and 2-DG treated samples (Fig. 4), one of our former studies did give rise to the assumption that the supplementation of pyruvate partially restored viral titers after glycolysis inhibition by 2-DG [10]. Since we wanted to further clarify the rescuing capacities of pyruvate addition, we first decided to investigate the effects on cellular energy metabolism during treatment with 2-DG with or without pyruvate. We performed real-time Seahorse-Analyzer measurements to examine the impact of pyruvate supplementation on glycolysis and cellular respiration during glycolysis inhibition (Fig. 5) to get insights into pyruvate mediated changes of host cell energetics under 2-DG treatment.

**Fig. 6:**
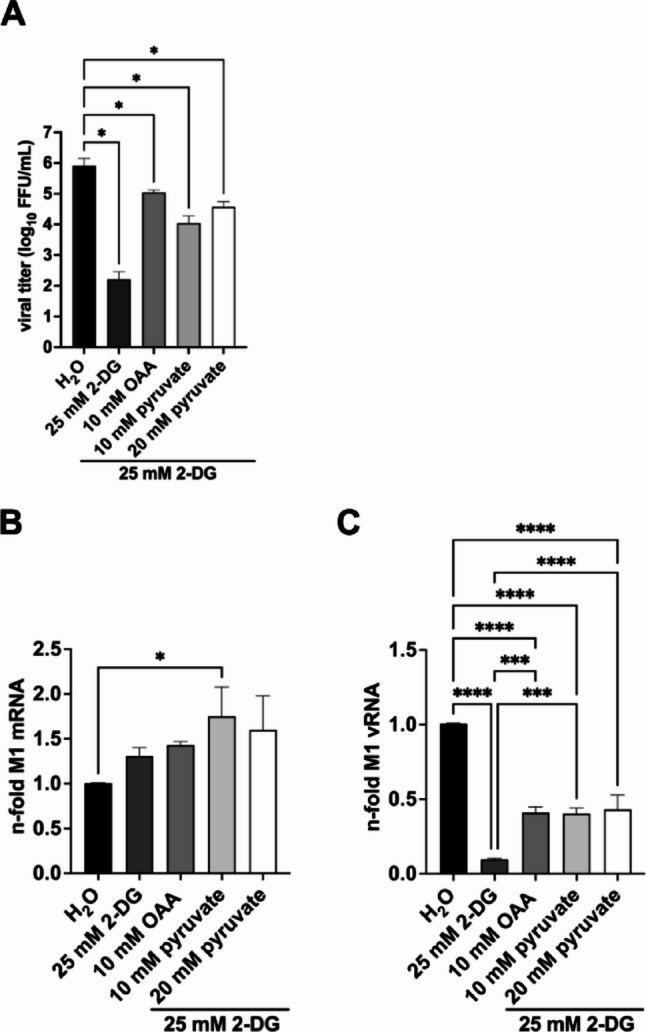
Similar to OAA, pyruvate exhibits a virus-rescuing effect under glycolysis inhibition. 24 h after seeding A549 cells, cells were infected with SC35M at an MOI of 0.001 (**A**) or 5 (**B**,** C**) for 30 min. Afterwards, the infected cells were treated either with 25 mM 2-DG, 10 mM oxaloacetate (OAA), or the indicated concentrations of pyruvate in combination with 25 mM 2-DG, or the solvent control water in DMEM infection media for a total of 24 (**A**) or 8 h (**B**,** C**). (**A**) After an infection time of 24 h, viral titers were determined via focus forming assay in focus forming units per mL (FFU/mL). Depicted are the means ± SD of three independent experiments with biological triplicates per condition and experiment. For real-time qPCR, RNA was isolated and cDNA was synthesized using either oligo(dT) primers to transcribe mRNA **(B)** or fluA uni12 primers to transcribe vRNA **(C)**. Realtime qPCR was performed with two technical replicates per sample, and values of the treated samples were normalized to the water control. In case of mRNA detection, all results were additionally normalized to a GAPDH control. Depicted are the means ± SD of three independent experiments with biological triplicates per condition and experiment. Statistical significances were determined via ordinary ANOVA with Tukey’s correction where all samples were compared to each other. P-values are indicated as follows: < 0.05 = *, < 0.01 = **, < 0.001 = ***, < 0.0001 = ****

Hence, we first measured the glycolytic proton efflux rate (glycoPER), extracellular acidification rate (ECAR), and oxygen consumption rate (OCR) under basal conditions followed by the injection of 25 mM 2-DG, either together with or without 20 mM pyruvate. The glycoPER and ECAR values serve as indicators of glycolytic activity, whereas the OCR reflects cellular respiration. Samples treated with 25 mM 2-DG revealed a massive decrease of glycoPER and ECAR, which aligns with earlier findings [10]. If pyruvate (20mM) was added in addition to 2-DG, an even more pronounced decrease was observed for the glycoPER and ECAR levels (Fig. 5A, B). For OCR, we detected a considerable increase with the addition of the glycolysis inhibitor and even an even higher OCR jump with the addition of pyruvate (Fig. 5C). In addition to these insights into pyruvate mediated changes in bioenergetics under glycolysis inhibition, we also tested the IAV growth rescuing capacities of pyruvate supplementation. For a direct comparison of the OAA and pyruvate rescue capabilities under 25mM 2-DG treatment we measured the number of newly produced infectious IAV particles produced by infected A549 cells (Fig. 6A).

We detected the expected reduction of viral titers under 2-DG treatment and the rescue of viral titers after addition of OAA. Moreover, 10 mM and 20 mM pyruvate supplementation resulted in virus rescuing capacities similar to those of OAA. Of note, we also supplemented infection media with pyruvate alone without any further 2-DG treatment to assess its direct effects on IAV growth and found no statistically significant alterations in the production of new infectious IAV particles compared with the control (Fig. S4).

Next, we also examined whether adding pyruvate affects the accumulation of viral mRNA and vRNA under 2-DG treatment after one replication cycle (Fig. 6B, C). Consistent with the restored viral titers observed after supplementing OAA or pyruvate under glycolysis inhibition, we found a partial recovery of vRNA levels compared with samples treated with 2-DG alone. The extent of this rescue was similar for both OAA and pyruvate. As observed previously, M1 mRNA levels increased in response to all treatments (Fig. 6B). Finally, to gain further insight into the underlying mechanisms of the observed phenotype, we analyzed the effects of OAA and pyruvate on antiviral innate immune gene expression under glycolysis inhibition. To make a long story short, the different treatments resulted only in minor changes of the measurable IAV induced ISG expression (Fig. S5).

In summary, our findings confirm that IAV replication strongly depends on the host cell’s metabolic pathways. We demonstrated that supplementation with the TCA cycle metabolite OAA reversed the inhibitory effect of strong glycolysis inhibition by 2-DG on viral replication, viral RNA synthesis, and viral protein accumulation. A similar rescue effect was also observed upon supplementation with mannose, a metabolite closely linked to glycolysis, and with pyruvate, the end product of glycolysis. Furthermore, mass spectrometry-based metabolomic analyses revealed distinct metabolic alterations across treatments, including a strong accumulation of pyruvate in OAA-supplemented samples. These findings suggest that the OAA-mediated rescue of viral replication is primarily due to the restoration of intracellular pyruvate levels—a conclusion further supported by the observed rescue of viral growth upon direct supplementation with pyruvate.

## Discussion

Viruses have evolved different strategies to modify biosynthetic pathways of the host cell. Understanding the interplay between host cell metabolism and virus infections is required, as metabolic intervention might be a promising option for new host-directed antiviral strategies. The aim of the study was not to immediately test and develop a certain new antiviral drug, but rather to establish new fundamental knowledge about the interaction between IAV and the host cell metabolism, in order to lay a foundation for further host-directed antiviral therapy research (Fig. S6).

Many studies have highlighted that IAV manipulates glucose metabolism to promote its own replication [8, 9, 10, 18]. As examples, it was already shown by us that inhibition of glycolysis with 2-DG strongly decreased IAV propagation in a dose-dependent manner [10]. Furthermore, we found that the decrease in viral replication observed after 2-DG treatment resulted from an inhibition of the viral polymerase–mediated synthesis of genomic vRNA, whereas the synthesis of viral mRNA was not statistically significantly altered [10]. This phenomenon of derailed IAV polymerase activity may be a consequence of more general effects of metabolic interference, as inhibition of glutaminolysis, FAS, and OXPHOS also led to statistically significantly reduced viral genomic RNA [19]. Consistent with all these treatments, we have observed that IAV polymerase activity correlates with an imbalance in cellular glycolysis and cellular respiration caused by the aforementioned inhibitors, leading to a decreased production of vRNA [19].

Since these metabolic pathways are all connected to glycolysis mostly through the TCA cycle, we aimed to gain a deeper understanding of how the metabolic network is altered during IAV infection under conditions of strong glycolysis inhibition and whether supplementation with intermediates of the TCA cycle would rescue IAV propagation. The rationale for this objective is based on the metabolic map where e.g. glucose is finally metabolized into acetyl-CoA and thereby inserted into the TCA. In our study we were able to expand our knowledge about effects of TCA cycle metabolic fueling intermediates in the life cycle of IAV during glycolysis inhibition. As numerous other studies, we confirmed the antiviral role of the potent glycolysis inhibitor 2-DG in IAV infected cells [10, 15, 30], here using 25 mM of 2-DG to manifest its impact on cellular metabolism and IAV replication. Since we detected the virus growth rescue capacities of the TCA metabolite OAA during glycolytic rate inhibition, it is noteworthy, that OAA is described as a key metabolite of the TCA cycle and not itself a glycolysis intermediate [25, 31]. While glycolysis is known to be critical for efficient IAV propagation, the TCA cycle also plays an essential role for viral replication as it remains active in infected cells [8, 9]. In the early 1950s, it was already reported that IAV propagation in ex vivo infected mice tissue was found to be inhibited by the addition of inhibitors of TCA cycle enzymes [32]. Viruses depend strongly on various metabolic substrates for efficient viral replication, however, for most of these substrates, including OAA, the specific molecular impact on viral life cycle has not been explored. Surprisingly, we could nearly rescue the antiviral effects of 2-DG by supplementation with 10 mM OAA in viral titer experiments (Fig. 1B, C). These virus promoting effects of the TCA metabolite OAA seems to be quite specific, given that citrate, another TCA cycle metabolite, has been reported to exert antiviral effects in infections [33, 34]. On the other hand, other metabolic products are known to have proviral effects too, such as lactate. For a long time, lactate was only described as a by-product of glucose metabolism but in fact its accumulation serves as a substrate for lactylation, which promotes the virus replication through targeting of viral and host genes [35, 36]. Moreover, lactate has been described to inhibit the release of type I interferons (IFNs) [37] and to enhance IAV replication by suppressing MAVS-dependent induction of type I IFNs in primary human airway epithelial cells [38]. This body of literature indicates that specific metabolites do have a molecular impact on viral life cycles and that specific metabolites such as lactate [38] or extracellular ATP [39] do have functions as signaling molecules or cellular signaling regulating agents.

To further evaluate the virus promoting effects of OAA during glycolysis inhibition, we added mannose to the experimental setup. Mannose, a C2 epimer of glucose, is known to be closely linked to glycolysis and to reverse the effects of 2-DG by refueling glycolysis [10]. Here, it was of particular interest to compare OAA, which does not have a known direct metabolic path to the upper part of the glycolysis pathway, to mannose, a metabolite closely linked to glycolysis. Strikingly, OAA and mannose reversed the effects of 2-DG on viral titers and vRNA levels to a similar extent (Fig. 2A, C, E). In line with the increased viral titers and partial rescued vRNA levels, a statistically significant increase for the IAV PA, M1, NP, and NS1 protein accumulation during OAA and mannose supplementation was measured (Fig. 3A-E). In addition, elevated mRNA levels were observed for the treatment of 2-DG and in combination with OAA or mannose compared to control (Fig. 2B, D), an observation that is consistent with our previous study [10] and confirms the 2-DG provoked dynamic dysregulation of the polymerase functions to be either active as a transcriptase or a replicase. It would be interesting to study whether similar effects can be seen with this treatment protocols in other respiratory viruses or if the detected regulations are IAV specific. Although the molecular mechanism of this IAV growth rescue is still unclear and the exact viral polymerase switching process is not fully understood yet, it is likely that different host factors are involved [40, 41]. Specifically, the IAV polymerase interacts with human ANP32 to facilitate the replication of the viral genome. Thus, ANP32 may be a critical host cell mediator of the observed effects on viral mRNA and vRNA reported here (Fig. 2B-E) [40, 41]. Indeed, the metabolic interference with 2-DG is known to affect the cellular glycolysis and respiration balance [19], that may impair host factor functions taken part in the viral polymerase regulation. A plausible explanation is that OAA bypasses the impairment of host cell factors required for proper regulation of the viral polymerase, thereby mitigating the interference with the IAV replication cycle and leading to a partial restoration of vRNA accumulation levels (Fig. 2C, E).

To gain a broader insight into the metabolic changes underlying the OAA-mediated rescue, we employed HILIC-MS/MS to investigate the metabolic alterations induced by OAA under glycolysis inhibition. During infection, we observed changes in the metabolic profile and an upregulation of certain metabolic intermediates, particularly glucose (Fig. 4) (Fig S3). Moreover, the combined treatment of OAA and 2-DG resulted in increases of certain TCA cycle intermediate levels, most notably acetyl-CoA and citric acid. Most prominent, the addition of OAA under glycolysis inhibition led to a marked and statistically significantly increase of pyruvate levels, independent of infection. Our hypothesis here is that cells may metabolize OAA to pyruvate during metabolic stress, resulting in compensation of the inhibited glycolytic rate. Interestingly, pyruvate concentrations were higher in uninfected cells treated with OAA and 2-DG than in infected cells subjected to the same treatment, indicating that viral infection promotes an increased utilization of pyruvate through downstream metabolic pathways (Fig S3). This finding is in line with the literature, which indicates that infected cells rely on aerobic glycolysis [14], a process where pyruvate is converted to lactate. However, the metabolic pathways described in the literature to date for the conversion of OAA to pyruvate involve a series of metabolic reactions and are not within a direct single step pathway [42, 43]. For example, OAA can be reduced to malate by malate dehydrogenase in the mitochondria. Malate is then exported into the cytosol, where it is converted to pyruvate or back to OAA, in which case the enzyme phosphoenolpyruvate carboxykinase (PEPCK) decarboxylates OAA to phosphoenolpyruvate (PEP). Finally, PEP is converted to pyruvate via pyruvate kinase, which is also the last step of glycolysis (Fig. S6) [43]. However, this metabolic pathway of OAA reduction to malate seems to be unlikely as an explanation for the elevated pyruvate levels in our samples, as malate is an obligate intermediate in this reaction and we were unable to detect elevated malate levels in the mass spectrometry analysis (Fig. 4).

Our results are consistent with previous studies demonstrating that OAA exerts pro-glycolysis effects, which are not attributable to spontaneous decarboxylation or enzymatic conversion of OAA to pyruvate [25]. While our data reveal a viral promoting effect of OAA supplementation during glycolysis inhibition in viral growth experiments, in a different experimental setting, previous studies have reported that OAA plays a role in host defense against viral infections [44]. Specifically, OAA supplementation has been shown to increase IFNβ production against IAV and thereby modulate antiviral innate immune responses against IAV [44]. However, we did not observe substantially altered ISG levels under OAA or pyruvate supplementation in the presence of 2-DG (Fig. S5). Notably, the addition of OAA alone did not affect the production of new infectious IAV particles compared to the control group (Fig. 1A). All together, these findings may highlight the complex role of immune and metabolic interactions in context of IAV infections, emphasizing the need for further research.

In our previous experiments, we demonstrated that upon inhibition of either glycolysis or cellular respiration rate, cells compensate for the loss by upregulating the respective alternative metabolic rate for producing energy (ATP) [10, 19]. Pyruvate is described to be the end product of glycolysis, subsequently will be transported to mitochondria to get further converted to acetyl-CoA [23]. Using Seahorse-based analysis to understand consequences of pyruvate supplementation to the bioenergetics of 2-DG treated cells, we recorded a substantial decrease of the glycoPER and ECAR and an increase of OCR. Of note, we measured an even higher OCR with the addition of pyruvate under glycolysis inhibition (Fig. 5C). While pyruvate supplementation was able to rescue viral replication under conditions of glycolysis inhibition, this effect seems not to stem from a reactivation of glycolysis. Finally, to assess the biological relevance of pyruvate levels in OAA-mediated rescue of IAV replication, we observed increased viral titers and enhanced vRNA accumulation in pyruvate-supplemented samples compared to those treated with 2-DG alone (Fig. 6). Interestingly, we could also show that pyruvate supplementation of infection media without 2-DG did not affect viral titers compared to control (Fig. S4). These findings are consistent with our previous reports demonstrating that pyruvate supplementation partially restores viral titers following inhibition by 2-DG [10]. Of interest, earlier studies have investigated the anti-inflammatory capacities of pyruvate [45, 46] and evidence suggests that supplementing pyruvate during IAV infection impairs cytokine production without inhibiting viral RNA replication [47].

In contrast, in our settings, data reveal that the OAA mediated rescue promotes IAV growth by providing intracellular pyruvate, which is obviously required for efficient IAV polymerase regulation and growth.

Although the conversion of OAA to pyruvate represents a reasonable mechanism for the observed pro-viral effects under metabolic stress, the question of the exact metabolic pathway from OAA to pyruvate remains open. While we can only speculate about the metabolic mechanism of OAA to pyruvate conversion, we were able to clearly demonstrate that the addition of pyruvate restores IAV replication during 2-DG treatment (Fig. 6). Key questions remain as to why glycolytic inhibition reduces viral genome replication while prolonging transcription, and how OAA supplementation partially restores vRNA levels. These findings suggest a functional dysregulation of the viral polymerase under glycolytic inhibition that is alleviated by the TCA cycle intermediate OAA. We propose that increased pyruvate levels help rebalance transcription and replication by partially compensating for reduced glycolytic flux. This effect may reflect altered cofactor availability, which is partially restored by OAA, potentially via enhanced pyruvate production (Fig. S7). The precise metabolic control of viral polymerase activity, however, remains to be defined.

Taken together, our study demonstrated the dependency of IAV on metabolic enzymes and metabolites for successful replication and increased the knowledge about host cell metabolism related virus-host interactions.

## Conclusion

This data support the assumption that specific metabolite intermediates are crucial for IAV replication. Our findings highlight the considerable promise of targeting host cell metabolism as an antiviral strategy. Our data demonstrate that the TCA cycle intermediate OAA has virus supporting effects as it reversed the antiviral effects of glycolysis inhibition. However, we still need to gain a more detailed understanding of the underlying mechanisms of metabolism-related virus-host interactions.

## Supplementary Information

Below is the link to the electronic supplementary material.

Supplementary Material 3: n-fold changes over control and statistical significances of Fig. 4.

Supplementary Material 4: Effects of pyruvate supplementation on viral growth. A549 cells were infected with SC35M at an MOI of 0.001 for 30 min. Cells were then incubated in DMEM infection media with 10 mM or 20 mM pyruvate or its solvent control water. After 24 h of infection, supernatants were collected and viral titers were detected via focus forming assay. Depicted are the means ± SD of three independent experiments with biological triplicates per condition and experiment. Statistical significances were determined via ordinary ANOVA with Dunnett’s correction where all samples were compared to the control. P-values are indicated as follows: < 0.05 = *, < 0.01 = **, < 0.001 = ***, < 0.0001 = ****.

Supplementary Material 5: Effects of OAA and pyruvate on antiviral innate immune gene expression under glycolysis inhibition. A549 cells were mock infected or infected with SC35M and then incubated with DMEM infection media containing 2-DG combined with treatments of OAA or pyruvate or water, which serves as a control for 8 h. Then, cells were lysed, their RNA was isolated, cDNA synthesized using uni12 primer to transcribe vRNA (A) and olido dT primer to transcribe messenger RNA (mRNA) (B, C). Real-time qPCR was performed with two technical replicates per sample and in case of mRNA detection, all results were normalized to a GAPDH control. Statistical significances were determined via ordinary ANOVA with Dunnett’s correction where all samples were compared to the control. P-values are indicated as follows: < 0.05 = *, < 0.01 = **, < 0.001 = ***, < 0.0001 = ****.

Supplementary Material 6: Schematic representation of the metabolic routes described in this study. This illustration depicts the dynamic interplay of the metabolic pathways glycolysis and the tricarboxylic acid (TCA) cycle in eukaryotic cells. The schematic representation highlights the mannose mediated rescue of glycolytic flux through mannose-6-phosphate isomerase (MPI). Shown are different metabolic routes by which pyruvate can be imported in the mitochondria, including direct import via the mitochondrial pyruvate carrier (MPC) or indirect entry via alanine. Pyruvate can then be converted to oxaloacetate via pyruvate carboxylase (PC) or to acetyl-CoA via the pyruvate dehydrogenase complex (PDH). Acetyl-CoA-derived mitochondrial malate is exported to the cytosol and then oxidized to oxaloacetate in the cytosol, which can subsequently be converted to phosphoenolpyruvate (PEP) by phosphoenolpyruvate carboxykinase (PEPCK). Finally, PEP is reduced to pyruvate via pyruvate kinase. Created with BioRender.com.

Supplementary Material 7: Schematic representation of the effects of OAA treatment and glycolysis inhibition on viral replication and transcription. Schematic overview of the impact of OAA treatment and glycolysis inhibition on the formation of new viral mRNA and vRNA from viral ribonucleoprotein (vRNP) complexes. Replication proceeds normally in infected cells, indicated by a bold black line (**A**), or is strongly reduced (light grey line, **B**), or is partially rescued (black line, not bold, **C**). Reduced vRNA formation reflects impaired viral genome replication in the presence of the metabolic inhibitior 2-DG, as well as potentially reduced availability of cellular and/or viral cofactors required for efficient vRNA synthesis. Created with BioRender.com.

Supplementary Material 2: Corresponding blots of Fig. 3. A549 cells were mock-infected or infected with SC35M at an MOI of 5 for 30 min. Afterwards, the infected cells were treated either with 25 mM 2-DG, or 2-DG combined with 10 mM oxaloacetate (OAA), or 1 mM mannose (man), or the solvent control water in DMEM infection media for a total of 8 h. Protein lysates were harvested, equal amounts of protein were separated via SDS-PAGE and subjected to Western Blot analysis. Visualization was done using primary antibodies against PA (rabbit) (**A**), M1 (mouse) (**B**), vinculin (mouse) (**C**) for blot 1 and NP (rabbit) (**D**), NS1 (rabbit) (**E**), and vinculin (mouse) (**F**) for blot 2 and fluorescence-labelled secondary antibodies. Western Blot images were cropped and representative blots out of three independent experiments are shown. Illustrated are all original blots used for Fig. 3.

Supplementary Material 1: Representative images of viral foci from FFA. Representative images show stained foci in MDCK cells infected with SC35M at serial dilutions from 10^1 to 10^4. Minor deviations from ideal 10-fold reductions of focus forming units in 10-fold serial dilutions may occur due to assay variability. Foci were stained in violet but are depicted in white in this figure to enhance clarity and visibility. They were automatically detected and quantified by Celigo Image Cytometer (Nexcelom/Perkin Elmer Inc., Waltham, MA, USA).

## Data Availability

All relevant data were within the paper and its Supplementary material files.
