# Supplementary figures and images for: The activation of the metabolic oxaloacetate-pyruvate axis restores influenza A virus replication during impaired glycolysis
Source: Virol J. 2026 May 24;23:131. doi: 10.1186/s12985-026-03201-6 (PMC13200417; doi:10.1186/s12985-026-03201-6)

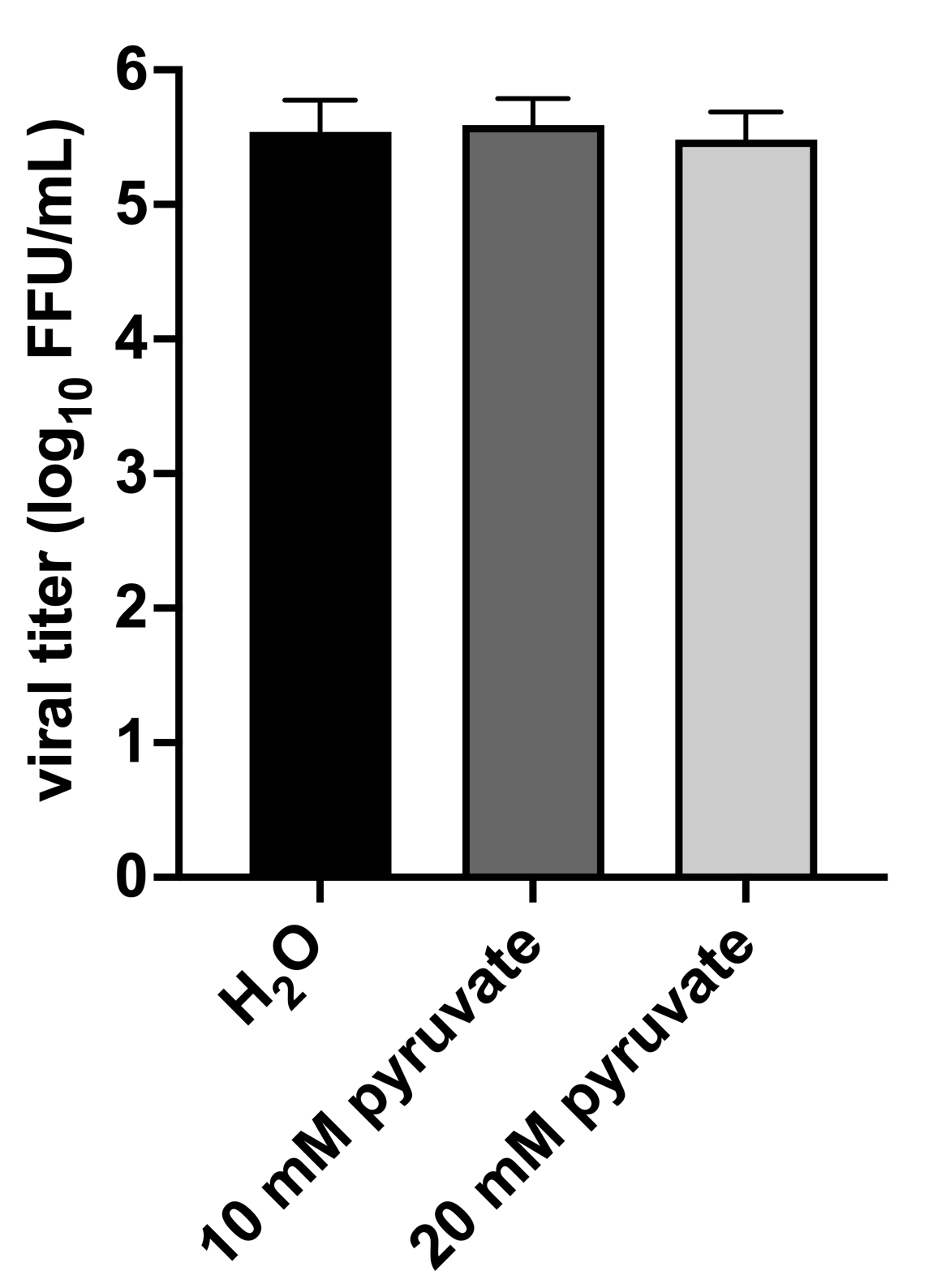

Supplement: Supplementary file 2 — Supplementary Material 4: Effects of pyruvate supplementation on viral growth. A549 cells were infected with SC35M at an MOI of 0.001 for 30 min. Cells were then incubated in DMEM infection media with 10 mM or 20 mM pyruvate or its solvent control water. After 24 h of infection, supernatants were collected and viral titers were detected via focus forming assay. Depicted are the means ± SD of three independent experiments with biological triplicates per condition and experiment. Statistical significances were determined via ordinary ANOVA with Dunnett’s correction where all samples were compared to the control. P-values are indicated as follows: < 0.05 = *, < 0.01 = **, < 0.001 = ***, < 0.0001 = ****. [file 12985_2026_3201_MOESM2_ESM.tiff]

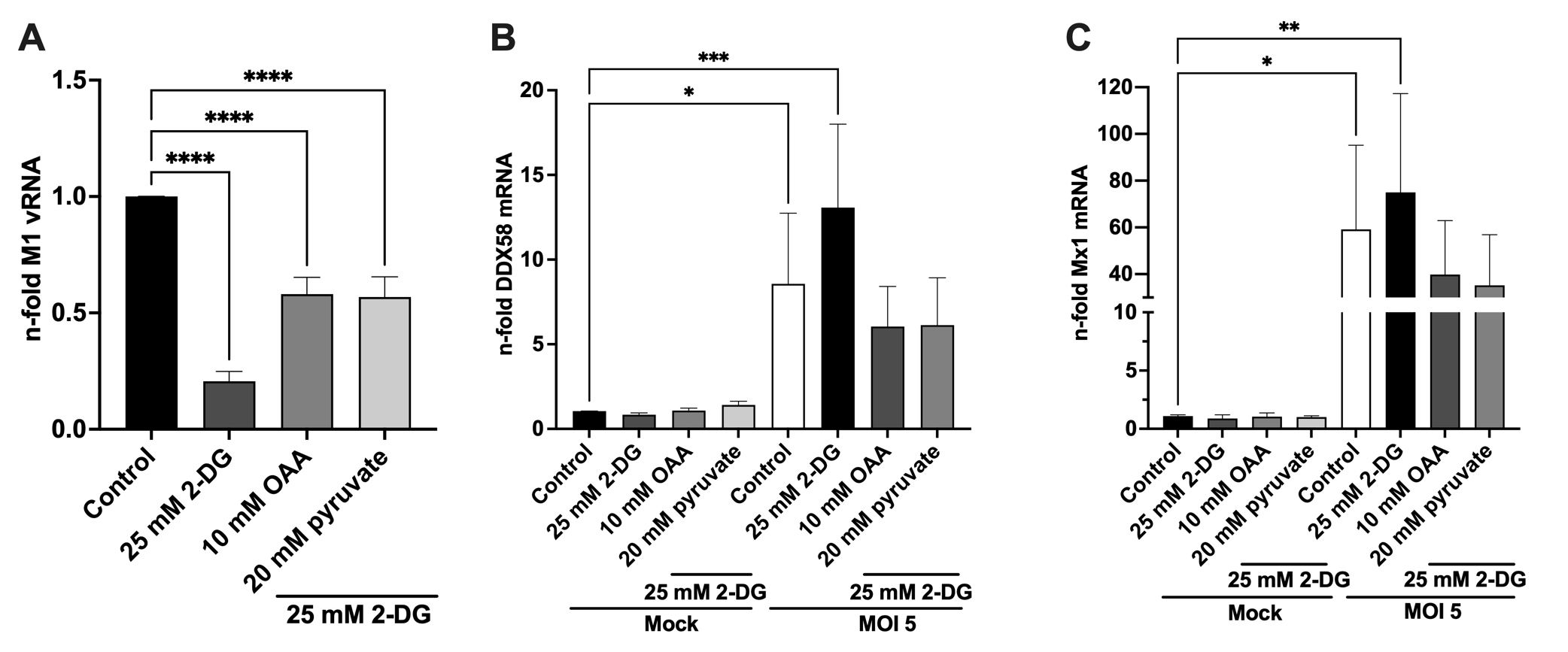

Supplement: Supplementary file 3 — Supplementary Material 5: Effects of OAA and pyruvate on antiviral innate immune gene expression under glycolysis inhibition. A549 cells were mock infected or infected with SC35M and then incubated with DMEM infection media containing 2-DG combined with treatments of OAA or pyruvate or water, which serves as a control for 8 h. Then, cells were lysed, their RNA was isolated, cDNA synthesized using uni12 primer to transcribe vRNA (A) and olido dT primer to transcribe messenger RNA (mRNA) (B, C). Real-time qPCR was performed with two technical replicates per sample and in case of mRNA detection, all results were normalized to a GAPDH control. Statistical significances were determined via ordinary ANOVA with Dunnett’s correction where all samples were compared to the control. P-values are indicated as follows: < 0.05 = *, < 0.01 = **, < 0.001 = ***, < 0.0001 = ****. [file 12985_2026_3201_MOESM3_ESM.tiff]

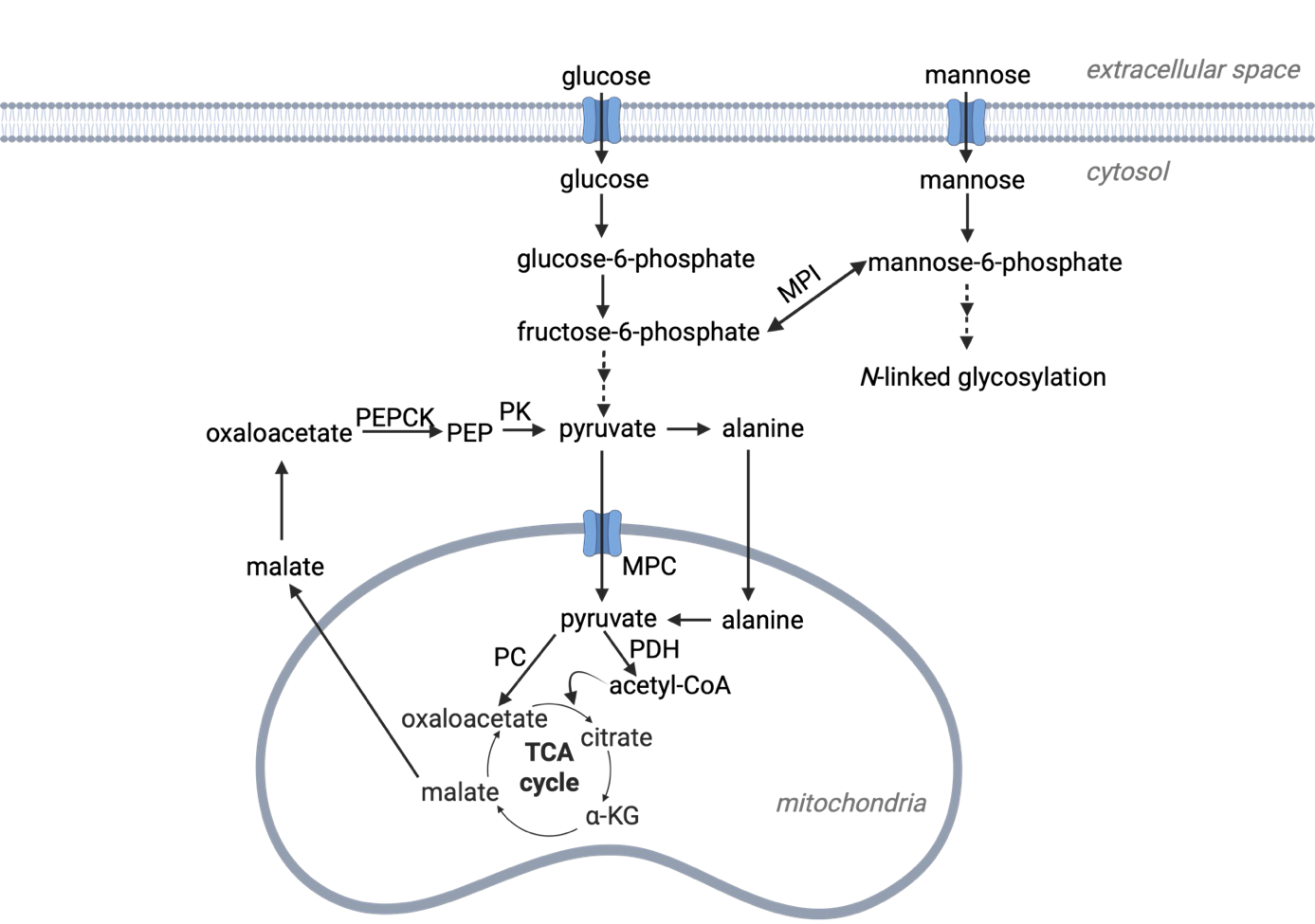

Supplement: Supplementary file 4 — Supplementary Material 6: Schematic representation of the metabolic routes described in this study. This illustration depicts the dynamic interplay of the metabolic pathways glycolysis and the tricarboxylic acid (TCA) cycle in eukaryotic cells. The schematic representation highlights the mannose mediated rescue of glycolytic flux through mannose-6-phosphate isomerase (MPI). Shown are different metabolic routes by which pyruvate can be imported in the mitochondria, including direct import via the mitochondrial pyruvate carrier (MPC) or indirect entry via alanine. Pyruvate can then be converted to oxaloacetate via pyruvate carboxylase (PC) or to acetyl-CoA via the pyruvate dehydrogenase complex (PDH). Acetyl-CoA-derived mitochondrial malate is exported to the cytosol and then oxidized to oxaloacetate in the cytosol, which can subsequently be converted to phosphoenolpyruvate (PEP) by phosphoenolpyruvate carboxykinase (PEPCK). Finally, PEP is reduced to pyruvate via pyruvate kinase. Created with BioRender.com. [file 12985_2026_3201_MOESM4_ESM.tiff]

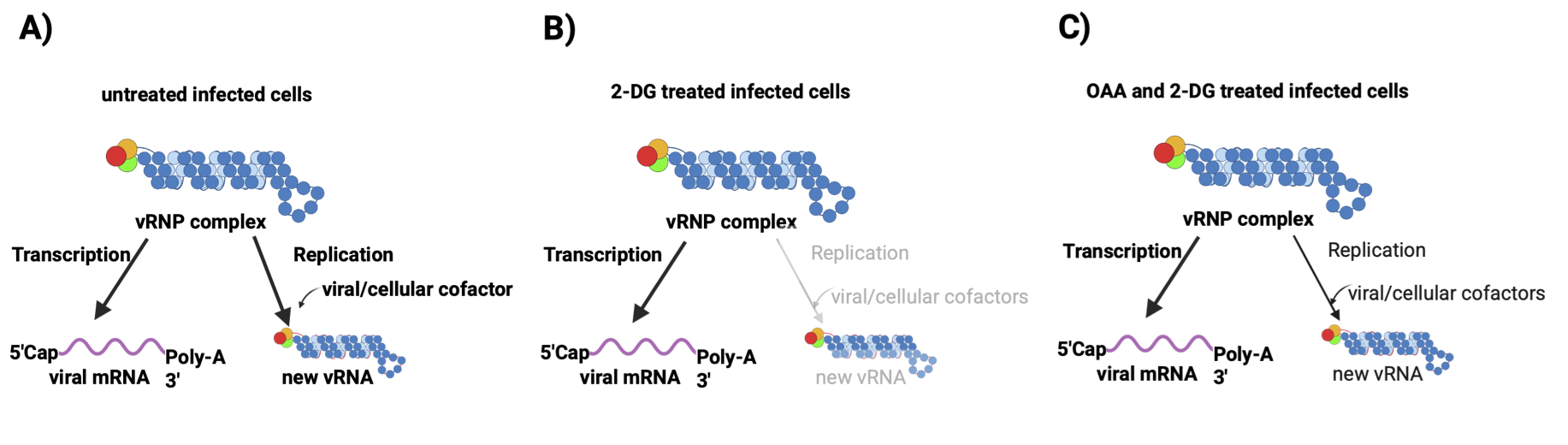

Supplement: Supplementary file 5 — Supplementary Material 7: Schematic representation of the effects of OAA treatment and glycolysis inhibition on viral replication and transcription. Schematic overview of the impact of OAA treatment and glycolysis inhibition on the formation of new viral mRNA and vRNA from viral ribonucleoprotein (vRNP) complexes. Replication proceeds normally in infected cells, indicated by a bold black line (A), or is strongly reduced (light grey line, B), or is partially rescued (black line, not bold, C). Reduced vRNA formation reflects impaired viral genome replication in the presence of the metabolic inhibitior 2-DG, as well as potentially reduced availability of cellular and/or viral cofactors required for efficient vRNA synthesis. Created with BioRender.com. [file 12985_2026_3201_MOESM5_ESM.tiff]

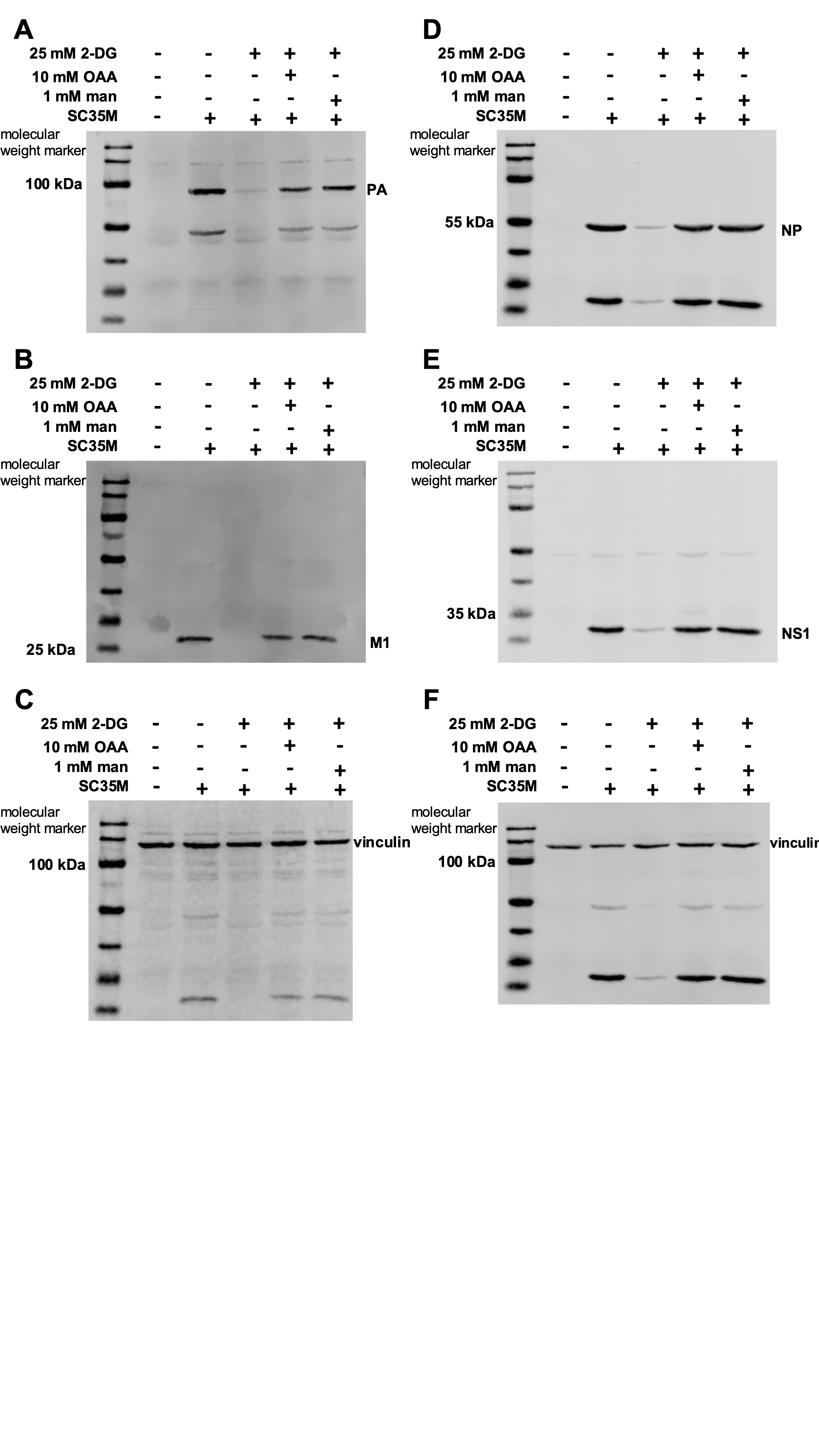

Supplement: Supplementary file 6 — Supplementary Material 2: Corresponding blots of Fig. 3. A549 cells were mock-infected or infected with SC35M at an MOI of 5 for 30 min. Afterwards, the infected cells were treated either with 25 mM 2-DG, or 2-DG combined with 10 mM oxaloacetate (OAA), or 1 mM mannose (man), or the solvent control water in DMEM infection media for a total of 8 h. Protein lysates were harvested, equal amounts of protein were separated via SDS-PAGE and subjected to Western Blot analysis. Visualization was done using primary antibodies against PA (rabbit) (A), M1 (mouse) (B), vinculin (mouse) (C) for blot 1 and NP (rabbit) (D), NS1 (rabbit) (E), and vinculin (mouse) (F) for blot 2 and fluorescence-labelled secondary antibodies. Western Blot images were cropped and representative blots out of three independent experiments are shown. Illustrated are all original blots used for Fig. 3. [file 12985_2026_3201_MOESM6_ESM.tiff]

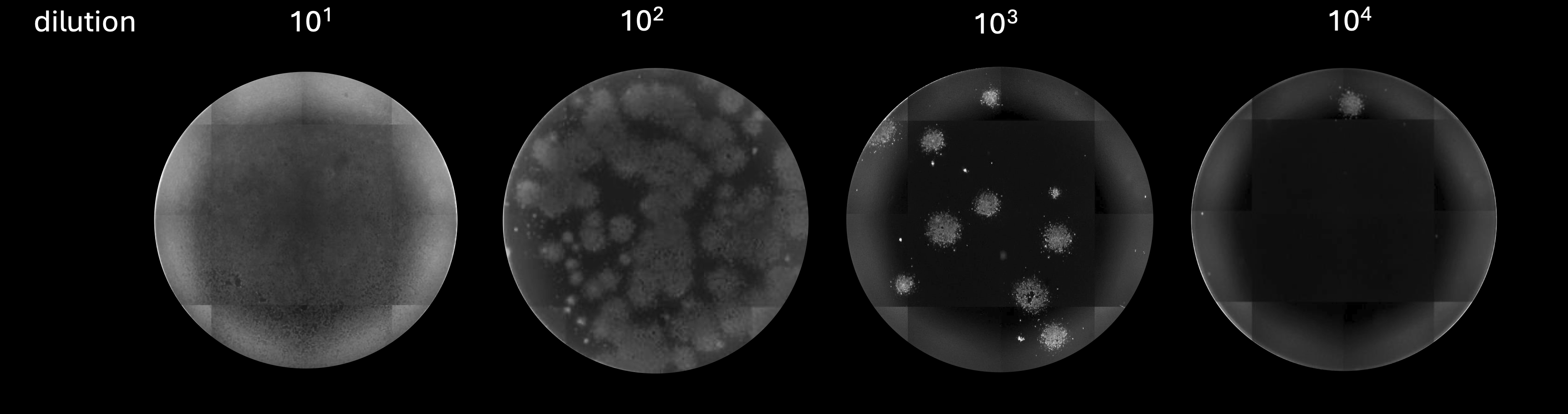

Supplement: Supplementary file 7 — Supplementary Material 1: Representative images of viral foci from FFA. Representative images show stained foci in MDCK cells infected with SC35M at serial dilutions from 10^1 to 10^4. Minor deviations from ideal 10-fold reductions of focus forming units in 10-fold serial dilutions may occur due to assay variability. Foci were stained in violet but are depicted in white in this figure to enhance clarity and visibility. They were automatically detected and quantified by Celigo Image Cytometer (Nexcelom/Perkin Elmer Inc., Waltham, MA, USA). [file 12985_2026_3201_MOESM7_ESM.tiff]
